# Human bipedalism and body-mass index

**DOI:** 10.1038/s41598-017-03961-w

**Published:** 2017-06-16

**Authors:** Su Do Yi, Jae Dong Noh, Petter Minnhagen, Mi-Young Song, Tae-Soo Chon, Beom Jun Kim

**Affiliations:** 1 CCSS, Department of Physics and Astronomy, Seoul National University, Seoul, 08826 Korea; 2 Department of Physics, University of Seoul, Seoul, 02504 Korea; 3 School of Physics, Korea Institute for Advanced Study, Seoul, 02455 Korea; 4 Department of Physics, Umeså University, SE-901 87 Umeå, Sweden; 5 Inland Fisheries Research Institute, National Institute of Fisheries Science, Gyeonggi-do, 12453 Korea; 6 Department of Biological Sciences, Pusan National University, Busan, 46241 Korea; 7 Ecology & Future Research, Association (EnFRA)y, Busan, 46228 Korea; 8 Department of Physics, Sungkyunkwan University, Suwon, 16419 Korea

## Abstract

Body-mass index, abbreviated as BMI and given by *M*/*H* ^2^ with the mass *M* and the height *H*, has been widely used as a useful proxy to measure a general health status of a human individual. We generalise BMI in the form of *M/H* ^*p*^ and pursue to answer the question of the value of *p* for populations of animal species including human. We compare values of *p* for several different datasets for human populations with the ones obtained for other animal populations of fish, whales, and land mammals. All animal populations but humans analyzed in our work are shown to have *p* ≈ 3 unanimously. In contrast, human populations are different: As young infants grow to become toddlers and keep growing, the sudden change of *p* is observed at about one year after birth. Infants younger than one year old exhibit significantly larger value of p than two, while children between one and five years old show *p* ≈ 2, sharply different from other animal species. The observation implies the importance of the upright posture of human individuals. We also propose a simple mechanical model for a human body and suggest that standing and walking upright should put a clear division between bipedal human (*p* ≈ 2) and other animals (*p* ≈ 3).

## Introduction

Lots of measurable traits of individual organisms are characterised by the Gaussian distribution, as expected from the central-limit theorem in statistics. The most convenient phenotypical traits to measure are physical sizes and weights of individuals. (Note that in the present paper we use the weight and the mass as interchangeable terms following the common usage of the two in public. In a more scientific terminology, the weight needs to be replaced by the mass throughout the paper.) For example, the height of a human individual, which is very easy to measure, is known to approximately follow the Gaussian distribution, and so is the weight. Quetelet in 1842 coined the term *normal man* in his book “ *A Treatise on Man and the Developments of his Faculties*” from the collection of data obtained from French soldiers^1^. The allometric relation *M* ~ *H* ^*p*^ between the mass *M* and the height *H* describes how the two quantities covary across individuals. For a given value of *H*, the average value of *M* follows the allometric relation *M* ~ *H* ^*p*^ and individual masses exhibit residual variations around the average. In ref. 2, it has been found that if we use *x* ≡ *M*/*H* ^2^ as a variable, the correlation between *x* and *H* becomes much weaker than when other integer values of *p* in *M*/*H* ^*p*^ are used. This interesting finding suggests that we can associate the Body-mass-index (BMI) *x* ≡ *M*/*H* ^2^ with health from an average perspective. If an individual has BMI far away from the average value, we would consider him unhealthy. However, it is very important to note that the concept of BMI as a general health measure is based only on statistics, and there is no rigorous physiological justification of the wide use of BMI. Nevertheless, the allometric perspective of BMI can serve as a nice starting point, especially when we do not know how to explicitly quantify metrics for health within a very complex physiology. After the birth of the concept of BMI, it has been used in a broad range of applications, and has become an everyday common sense knowledge in public. It has been found that the waist circumference^3^ and the blood pressure^4^ are strongly correlated with BMI.

It has been suggested that the value of *p* in *x* ≡ *M*/*H* ^*p*^ may not be exactly two, and the use of a noninteger value of *p* has been suggested by Benn^5^. The Benn index *p* has later been measured in different datasets, and a range of values around two have been obtained as summarised in ref. 6. It has also been found that the value of *p* depends on gender (male population usually has a larger value than female population)^7^, and adolescent populations have a significantly larger value of *p* ^8^. It has been recently reported that *p* ≈ 1.63 in Hong Kong and *p* ≈ 2.35 in Sweden better describe the real datasets^9^. When the exponent *p* = 3 is used in *M*/*H* ^*p*^, the value *x* = *M*/*H* ^3^ is called ponderal index (or corpulence index). The ponderal index has been used in the medical community as a measure of leanness, particularly for very young children. It has also been suggested that the small value around *p* ≈ 2 can be an artifact of an regression analysis applied for heavily scattered data and that the actual human body can be more isometric (*p* ≈ 3) than described by *p* ≈ 2^10^. Use of the sitting height or leg length rather than total height for *H* has also been suggested^11, 12^. In the present work, we aim to find the Benn index *p* for human and animals through the use of the linear regression method applied for each dataset: We plot $\mathrm{ln}\,M$ versus $\mathrm{ln}\,H$ and find the most suitable value of *p* in $\mathrm{ln}\,M\sim p\,\mathrm{ln}\,H+{\rm{const}}$. Alternatively, we can minimise the Pearson correlation between *H* and *M*/*H* ^*p*^ to get the value of *p* similarly to ref. 2.

## Animal Species

**Figure 1:**
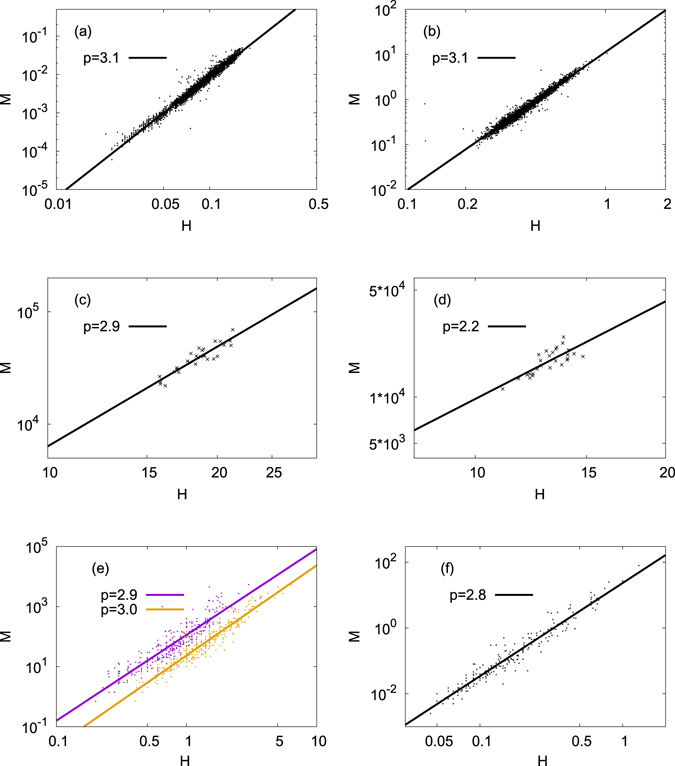
The mass *M* versus the linear body size *H* of animals: (**a**) Pale chub (*Zacco platypus*) (*N* = 3 163) and (**b**) Korean chub (*Zacco koreanus*) (*N* = 997) are for fishes, (**c**) Fin (*N* = 29) and (**d**) Sei (*N* = 27) are for whales, and (**e**) and (**f**) are for land mammals, respectively. The data file used for (**e**) contains all 325 lines of the mass, the body length, and the shoulder height information of many different species of land mammals, while the file used for (**f**) contains 205 lines of the mass and the body length of species in order Rodentia. In (**e**) two different ways to measure the linear size, the shoulder height (upper points) and the body length (lower points) are displayed. In all datasets in (**a**)–(**f**), we observe that the Benn index *p* ≈ 3, with (**d**) an exception, probably due to insufficient data size for Sei whales.

We start from the analysis of datasets (Supplementary Dataset 1) provided by National Institute of Fisheries Science in Korea for four different fish species of Pale chub (*Zacco platypus*) (*N* = 3 163), Korean chub (*Zacco koreanus*) (*N* = 997), Pond smelt (*Hypomesus nipponensis*) (*N* = 504), and Bastard halibut (*Paralichthys olivaceus*) (*N* = 3 077), with *N* being the number of individual fishes in each dataset. The body length of a fish is called *H* and the mass *M*. We find the Benn index *p* for each dataset through the use of the linear regression method. Almost the same value of *p* is obtained when we minimise the Pearson correlation between *H* and *M*/*H* ^*p*^. Suppose that we have a set of isometric three-dimensional creatures, with the mass density of each individual being almost constant. Just like a cube with the twice longer linear size has the eight times larger volume, we expect that the mass of a creature must be proportional to *H* ^3^, which yields the Benn index *p* = 3 for a three-dimensional scale-invariant creatures. In Fig. 1(a) and (b), we display the scatter plots for *M* versus *H* for Pale chub and Korean chub, respectively. It is clearly shown that the Benn indices for both fish species are given by *p* ≈ 3. As described above, the Benn index *p* = 3 implies that fishes are isometric and that doubling the size of each dimension does not change the shape of fish. Although not shown here, we find almost the same value (*p* ≈ 3) for other two fish species, Pond smelt and Bastard halibut.

Our result of the Benn index close to three for fish is significantly different from what are known for human populations. One possible conjecture to explain the difference is that human and fish belong to different classes, i.e., mammal and fish. Other possible explanation is that humans are land animal and fish live in water. Sea mammal like whales can then be a proper choice to compare. A theoretical study on the size limit of whales can be found in ref. 13. The empirical data for whales we use in the analysis contain relatively small number of individuals^14^, i.e., (c) *N* = 29 for Fin whales and (d) *N* = 27 for Sei whales in Fig. 1. It is difficult to draw a clear conclusion due to the small data sizes. Nevertheless, we point out that the regression result for Fin whales in Fig. 1(c), which appears to be of better quality than for Sei whales in Fig. 1(d), is consistent with *p* ≈ 3 similarly to the above result for fish. Accordingly, we may conclude that the well-known value *p* ≈ 2 for human populations is not because humans are mammals, but probably reflects the fact that humans live on land and that fish and whales live in water, in which the effect of gravity is much weaker due to the buoyant force. The next step to pursue is to compare with land mammals other than human.

For the analysis to get the Benn index for land mammals, we use two different datasets from ref. 15. Differently from other datasets used in the present paper which contain *intraspecific* variations, the datasets from ref. 15 contain *interspecific* variations and thus each value in the datasets is the average value for a given species. The first set in ref. 15 contains the mass, the shoulder height, and the body length of various species of placental land mammals. The second set contains the mass and the body length data for other species of order Rodentia, for which the shoulder height appears to be hard to measure. For our aim to resolve the difference between humans and other animals, the dataset for mammal species with both the shoulder height and the body length is particularly interesting. We have already shown above that the smaller value of *p* for humans does not originate from the difference between mammals and fish, and conjecture that the difference might be due to the difference in habitat, land and water, pointing out the role played by the gravity. If the gravity plays an important role in determining the Benn index, the shoulder height and the body length can be related with the mass in distinctly different ways: the shoulder height is a length scale along the direction of the gravity but the body length of land mammals is in perpendicular direction to the gravity. Surprisingly, we find in Fig. 1(e) that the Benn indices obtained for the shoulder height and the body length are not much different and the both values are very close to *p* ≈ 3, which nullifies our above conjecture. Not surprisingly any more, we confirm the observation of *p* ≈ 3 also for species in order Rodentia in Fig. 1(f) for the mass and the body length relation. In summary of this section, we have shown that the significantly small value of the Benn index for humans is neither from that humans are mammal nor that humans are land animals. In order to improve the analysis for land mammals, it would be better to check the validity of *p* ≈ 3 also for ontogenetic and intraspecific datasets for a given land mammal species, which can be done in the future if reliable datasets are available. We expect that there is similarity between ontogenetic and interspecific values of *p* as a metabolic scaling exponent in ref. 16. The next conjecture we can make is that the significantly small Benn index of humans stems from that humans are bipedal and other land mammals analyzed in this section are quadrupedal.

## Humans

It is well known that the Benn index for human adults and adolescents is significantly smaller than 3^2, 8, 9^. From the results in the previous section we arrive at the prediction that bipedalism of human can be an important factor to explain the Benn index of humans. Interestingly, we note that we humans are not always bipedal across the whole life time but becomes bipedal after a certain period after birth. We in this section focus on datasets collected in Sweden (*N* = 37702) (Supplementary Dataset 2) and in Korea (*N* = 68989) which contain weights and heights of children with ages less than seven (Sweden) and five (Korea) years. (See Additional information.) In addition, we also use the dataset provided by World Health Organization (WHO) which contains the longitudinal daily average values of the mass and height of 8440 children as they grow till 1856 days after birth.

**Figure 2:**
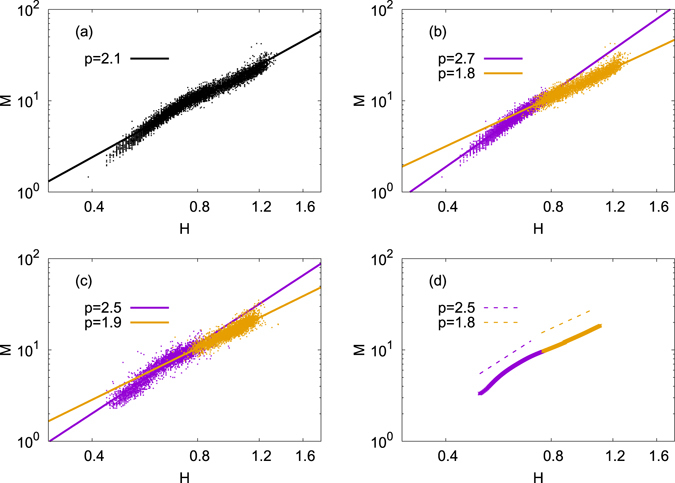
The mass *M* and the height *H* for humans: (**a**) and (**b**) for the data from Sweden, (**c**) from Korea, and (**d**) from WHO (see text for details of the used datasets). The linear regression for the entire data in (**a**) for Swedish children gives the Benn index *p* = 2.1. In (**b**), we divide all Swedish data into two groups depending on whether the child is younger or older than one year after birth. The left part of the data for children younger than one year old has *p* ≈ 2.8 while children older than one year (the right part of the data) has *p* ≈ 1.8. (**c**) Data for Korean children drawn in the same way as for (**b**), giving us *p* ≈ 2.5 and *p* ≈ 1.9 depending on the age group (younger and older than one year, respectively). (**d**) WHO data displayed in the same way. Again, we see the change of *p* value at around one year after birth. Although all data points in each dataset are used to perform the linear regressions in (**a**)–(**c**), we use only 10000 randomly chosen points in each scatter plot for (**a**)–(**c**), only for convenience of visibility.

Let us first focus on the dataset of Sweden. As seen in Fig. 2(a), the Benn index is far from 3 but close to 2, supporting previous studies^2, 8, 9^. The significantly smaller value of *p* ≈ 2 indicates that as a child grows, the growth in waist circumference is not as fast as the height, implying the strong influence of gravity of the Earth. Closer examination of Fig. 2(a) makes us recognise that there is a slight curvature around the middle part of the data. In order to find the reason of the existence of the curvature, we classify all children in the dataset in terms of the days after the birth, and obtain Fig. 2(b) from the same dataset as in Fig. 2(a). In Fig. 2(b), the left (right) part of the data points is for children of the ages less (larger) than 1 year. One can recognise clearly that a change of the value of the exponent occurs around at one year, which divides younger and elder children each characterised by the Benn index 2.7 and 1.8, respectively. We interpret this interesting change that occurs at around one year after birth as follows: Very young infants can neither stand up nor walk and thus we expect their growth is not strongly influenced by the direction of gravity. Consequently, infants in this age group exhibit the larger value *p* ≈ 2.7 ≈ 3 as obtained from fish in Fig. 1(a) and (b). As they grow to become toddler passing their one year birthdays, babies begin to stand and walk, and they become like adults in terms of posture, which explains the significantly smaller value *p* ≈ 1.8 ≈ 2 obtained in previous studies^2, 8, 9^. From the same reasoning, we also expect that a fetus in the mother’s womb also should exhibit *p* ≈ 3. In Fig. 2(c), we present our analysis of Korean data in the same way as in (b). We again find that infants of the age less than one year are sharply different from elder children of the age larger than one year: the former exhibits *p* ≈ 2.5 and the latter *p* ≈ 1.9. We then continue to analyze the dataset downloaded from the WHO website and show our result in Fig. 2(d). Again confirmed is that humans change their values of the Benn index *p* when babies pass through their one year birthdays, from *p* ≈ 2.5 to *p* ≈ 1.9. We believe that the observed change of *p* from datasets for Sweden, Korea, and WHO, unanimously support our conjecture that the upright posture of human adults and adolescents is the very reason of the difference between humans and other animals.

## Human bipedalism

In this section, we try to suggest an explanation why human populations except for infants exhibit the Benn index close to 2, in a sharp contrast to other animal populations. From the analysis in previous section for human children, it is very likely that the upright posture of humans is the origin of the small value of *p*, implying the importance of the gravity effect of the Earth. For a human to stand bolt upright against the gravity, the amount of muscle must be sufficiently large.

**Figure 3:**
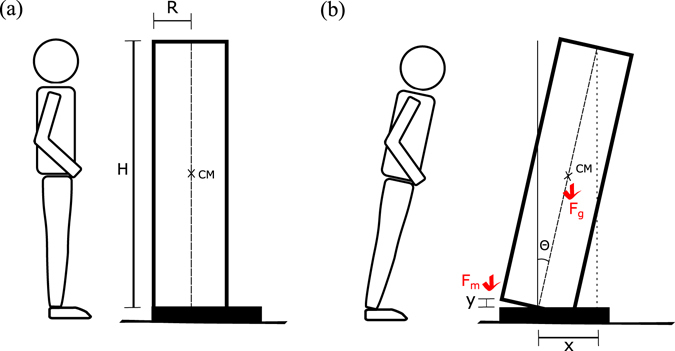
(**a**) We assume that the human body is a uniform cylinder with the radius *R* and the height *H*. It is supported on a rigid substrate (a representation of human feet). Suppose that the human body cylinder is tilted forward as shown in (**b**) by a small angle *θ*. To achieve the stability of the tilted posture, two competitive torques, one from the skeletal muscle force *F*_*m*_ and the other from the gravitational force *F*_*g*_, must be balanced. The gravitational torque is written as *T*_*g*_ = *F*_*g*_(*x*/2) with *x*/2 being the shift of the horizontal position of the center of mass (CM). We assume that *F*_*m*_ depends on the extension *y* of muscles as shown in (**b**). For the tilting angle *θ*, we get $x=H\,\sin \,\theta$ and $y=R\,\sin \,\theta =xR/H$.

Only for convenience, let us consider a human body as a uniform cylinder with the radius *R* and the height *H* supported on a rigid substrate (we call this simplified model of a human body as the human body cylinder from now on). The substrate represents the feet and is coupled with the main body through a system of skeletal muscles [see Fig. 3(a)]. We emphasize that the situation we are mimicking is when a human body is tilted forward by a small angle. If the tilting angle becomes larger, a human eventually moves one step forward to keep balance. Accordingly, our model must be restricted to the case when the tilting angle is sufficiently small. The actual human body is not rigid; in our simplified model the body cylinder part includes main human body and two legs as one rigid body and the feet are fixed on ground. The only possible motion in this situation is the forward tilting by the angle *θ* [see Fig. 3(b)].

The total weight of the body is written as *F*_*g*_ = *πρR* ^2^ *Hg* with the mass density *ρ* and the gravitational acceleration *g*. As seen in Fig. 3(b), suppose that the body cylinder of a human is tilted forward in such a way that the top of the body is now shifted horizontally by the distance *x*. The gravitational torque, with the contact point of the body axis and the ground [see Fig. 3(b)] taken as the origin, reads

1 $${T}_{g}={F}_{g}\frac{x}{2},$$

since the center of mass (CM) is horizontally shifted by the distance *x*/2. Upon tilting, the body cylinder is lifted from the substrate, which results in the extension *y* of the muscle as displayed in Fig. 3(b). The force *F*_*m*_ caused by the extension of the muscle then applies the torque

2 $${T}_{m}={F}_{m}cR$$

with *c* being a constant of *O*(1). Skeletal muscles are collections of long muscle fibers, which are composed of repeating sections of the so-called sarcomeres. We expect that the total number of muscle fibers is proportional to *R* ^2^ and that the number of repeating building blocks (sarcomeres) along the muscle fiber must be proportional to *H* ^17^. Accordingly, we believe that the muscle force *F*_*m*_ which can be provided by the human body cylinder can roughly be written as

3 $${F}_{m}={R}^{2}Hf(y),$$

where *f*(*y*) is independent of body size but depends only on the extension *y* of the muscle. We note that in the study of jump heights of various animal species^18^ the available energy produced by a body has been argued to be proportional to the body mass, i.e., *R* ^2^ *H*, as in Eq. (3). The stability requirement for the tilted posture is given by the torque balance condition *T*_*g*_ = *T*_*m*_, which yields (*π*/2)*ρR* ^2^ *Hgx* = *cR* ^3^ *Hf*(*y*), and we obtain *x* ~ *Rf*(*y*). We then use a general form *f*(*y*) ~ *y* ^*b*^ with an unspecified parameter *b*, and get *x* ~ *Ry* ^*b*^. The final step is to use $x=H\,\sin \,\theta$ and $y=R\,\sin \,\theta =Rx/H$ as shown in Fig. 3, which gives us *x* ~ *R*(*Rx*/*H*)^*b*^. In order to make this equation self-consistent, we now find *b* = 1, and we finally get

4 $$H\sim {R}^{2},$$

yielding the Benn index *p* = 2 from *M* ~ *R* ^2^ *H* ~ *H* ^2^ for upright human body. Although our model results in *p* = 2 in accord with observations, we still believe that the model demands improvement to be more realistic. For example, the upper body length may scale differently than the total height *H*, which could be related with different values of *p* for different human population groups.

## Summary and Discussion

In summary, we have tried to answer the question why the human body-mass index has the form *M*/*H* ^2^. From the body length and the mass information of individual fishes of several different fish species, we have first shown that the Benn index *p* in *M*/*H* ^*p*^ for fish species is very close to three, in a sharp contrast to *p* ≈ 2 for humans. We have made the first conjecture that the difference between human (*p* ≈ 2) and fish (*p* ≈ 3) may arise because the former is mammal. The conjecture has been rejected from our analysis of sea mammals: Fin whales exhibit *p* ≈ 3 although they are mammals. These analyses for fish and whale have led us to make the second conjecture that the reason why humans have small value of *p* could be from the difference in habitat, i.e., humans live on land but fishes and whales in water. In order to check the validity of the second conjecture we have analyzed the quadrupedal land mammals. Surprisingly, the second conjecture has also been rejected from our analyses of land mammals. The body length and the mass of various species in order Rodentia are characterised by *p* ≈ 3. Even when the shoulder heights are used instead of the body lengths for other different species of land mammals, we have again found *p* ≈ 3. This is particularly interesting result in view of the directional difference between the shoulder height and the body length of quadrupedal land mammals: The former is measured along the direction of gravity, and the latter is in perpendicular direction to the gravity. The last possible conjecture for the uniqueness of the human body-mass index one can think of is then that it must be due to the fact that humans are bipedal, standing and walking upright. We have examined the last conjecture by comparing human individuals in different age groups, and have found the interesting difference in the value of *p* between infants younger than one year old (*p* ≈ 3) and children older than that (*p* ≈ 2). The difference is particularly important since young infants begin to walk to become toddlers approximately after one year birthdays. The value *p* = 2 clearly indicates that as a human grows in height *H*, the circumference *C* of the waist does not grow as fast as the height: If the two length scales grow in the same fashion (*C* ~ *H*), humans must have *p* = 3 since the body mass is proportional to *HC* ^2^ ~ *H* ^3^ in this case. In other words, if the value *p* = 2 for humans is correct, this indicates that the waist circumference of a human body needs to be proportional to the square root of the height, suggesting that the taller a person is the thinner (s)he should look. The slower growth of the waist circumference with the height *H* also suggests the importance of the gravity: As one grows taller, it is harder to stand and walk bolt upright in the gravitational field. We have then adopted a very simple mechanical toy model for a human body, and applied the balance condition for the gravitational torque and the torque provided by the muscle force, which have led us to our expected value *p* = 2 for a human body. To summarise our empirical analyses of various datasets, we present all our obtained Benn indices in Table 1.

**Table 1:** Summarised results of Benn indices *p* for all datasets used in the paper. It is clear that human and other animals have different values of *p*. While *p* ≈ 3 for young infants is close to *p* for other animals, the value drops down to *p* ≈ 2 after one year.

	species		*N*	*p*
fish	Pale chub (*Zacco platypus*)		3163	3.1
	Korean chub (*Zacco koreanus*)		997	3.2
	Pond smelt (*Hypomesus nipponensis*)		504	3.0
	Bastard halibut (*Paralichthys olivaceus*)		3077	3.1
whale^14^	Fin		29	2.9
	Sei		27	2.2
mammal^15^	placental mammals		325	2.9^*^
				3.0^†^
	Rodentia		205	2.8
human (children)	Sweden	<1 year	22710	2.7
		>1 year	14992	1.8
	Korea	<1 year	46070	2.5
		>1 year	22919	1.8
	WHO^19^	<1 year	—	2.5
		>1 year	—	1.9

*Shoulder height, ^†^body length.

Information on body size would be also useful for practical applications in ecology including productivity estimation and energetic processes^20, 21^. Insects and macroinvertebrates have been a good target in estimating the length-mass relationships, considering economic importance and availability of the large number of individuals by sampling as well. In aquatic macroinvertebrates, the Benn index *p* around 3 in *M* ~ *H* ^*p*^ were reported and *p* around 2 were observed for the larvae of aquatic insects with relatively flattened shapes^22, 23^. The study of the length-mass relation could be further conducted to provide in depth information on organisms’ survivability in adapting to surrounding environmental constraints according to topography of aquatic ecosystems (e.g., water velocity, substrate compositions). The index regarding body size would be also useful for estimating biodiversity. By measuring major orders in insect communities, Siemann *et al*.^24^ reported the highest levels in species richness and number of individuals were observed with distinct body size in the intermediate level (i.e., not in small size) in each order. In addition, the parameter relating to species richness and the number of individuals was stable. In the function, *S* ~ *I* ^*d*^, where *S* is species richness, and *I* is the number of individuals, the parameter *d* ≈ 0.5 was found to be stable across different size classes. In this case, however, the size class was only determined by biovolume, average product of length, width and thickness. By presenting the ratio between body mass and height with BMI, animals’ behaviour adaptation (e.g., swimmer, burrower, clinger) could be more specifically addressed under specific environmental conditions (e.g., riffle, pool) in aquatic macroinvertebrates in streams, for instance. Further studies are warranted in this regard.

## Sources of datasets

Data for fish species are provided by National Institute of Fisheries Science in Korea and included as Supplementary Dataset 1. Data for Swedish children are obtained by special permission for research purposes from Västerbottens County Council, Sweden, through P. Minnhagen and included as Supplementary Dataset 2. Data for Korean children can be provided by Ministry of Health and Welfare of Korea upon formal request.

## Electronic supplementary material

Dataset 1A

Dataset 1B

Dataset 1C

Dataset 1D

Dataset 2
